# Optimal surveillance strategies for bovine tuberculosis in a low-prevalence country

**DOI:** 10.1038/s41598-017-04466-2

**Published:** 2017-06-23

**Authors:** Kimberly VanderWaal, Eva A. Enns, Catalina Picasso, Julio Alvarez, Andres Perez, Federico Fernandez, Andres Gil, Meggan Craft, Scott Wells

**Affiliations:** 1 Department of Veterinary Population Medicine, University of Minnesota, 1365 Gortner Avenue, St. Paul, MN, 55108 USA; 2 Division of Health Policy and Management, School of Public Health, University of Minnesota, 420 Delaware Street SE, MMC 729, Minneapolis, MN 55455 USA; 3 Animal Health Bureau, Ministry of Livestock, Agriculture, and Fisheries, 1476 Constituyente, Montevideo, 11200 Uruguay; 4 Facultad de Veterinaria, Universidad de la Republica, 1550 Alberto Lasplaces, Montevideo, 11100 Uruguay

## Abstract

Bovine tuberculosis (bTB) is a chronic disease of cattle that is difficult to control and eradicate in part due to the costly nature of surveillance and poor sensitivity of diagnostic tests. Like many countries, bTB prevalence in Uruguay has gradually declined to low levels due to intensive surveillance and control efforts over the past decades. In low prevalence settings, broad-based surveillance strategies based on routine testing may not be the most cost-effective way for controlling between-farm bTB transmission, while targeted surveillance aimed at high-risk farms may be more efficient for this purpose. To investigate the efficacy of targeted surveillance, we developed an integrated within- and between-farm bTB transmission model utilizing data from Uruguay’s comprehensive animal movement database. A genetic algorithm was used to fit uncertain parameter values, such as the animal-level sensitivity of skin testing and slaughter inspection, to observed bTB epidemiological data. Of ten alternative surveillance strategies evaluated, a strategy based on eliminating testing in low-risk farms resulted in a 40% reduction in sampling effort without increasing bTB incidence. These results can inform the design of more cost-effective surveillance programs to detect and control bTB in Uruguay and other countries with low bTB prevalence.

## Introduction

In many countries, bovine tuberculosis (bTB) causes substantial economic losses due to costly surveillance, culling of infected animals, and imposition of movement restrictions in affected regions^1, 2^. The disease also represents a major public health concern, particularly in developing economies and rural regions due to transmission to farm workers and consumption of unpasteurized milk^3^. Prerequisite for the design and implementation of bTB surveillance systems is their ability to detect infection in cattle as early as possible to minimize spread and to mitigate costs of control and eradication^4^. Active bTB surveillance programs are costly and are complicated by limited sensitivity and specificity of diagnostic tests used to detect infected animals. In regions or countries with low prevalence, adopting risk-based (targeted) surveillance may improve the cost-effectiveness of bTB management compared to conventional surveillance strategies. Risk-based surveillance focuses on the subset of the population with a higher risk of infection, thus improving surveillance system sensitivity and reducing funding and labor investments^5^.

A primary risk factor for bTB transmission is the introduction of infected cattle into herds through cattle movements^6, 7^. Spread of bTB via animal movements is particularly important in areas with low bTB incidence^8–12^. However, to optimize the implementation of surveillance and control measures, additional research is needed to clarify which herds and locations are associated with higher risk for disease introduction and transmission, and to develop methods to identify high-risk herds at an early stage of infection. Animal traceability systems, which have been implemented in many countries, provide an ideal opportunity to empirically assess movement-related bTB risk and to simulate the potential between-farm spread of bTB through the cattle industry^11–14^.

Social network analysis (SNA) has been used to characterize patterns of cattle movement, quantify the role of high-risk farms, and assess the vulnerability of livestock industries to epidemics in a variety of countries^7, 15–21^. For example, following the 2001 Foot-and-Mouth Disease (FMD) epidemic in the UK, between-herd cattle movements were heavily scrutinized for their role in facilitating disease spread^22–24^. SNA provided a framework to assess the importance of these movements, develop mathematical models to predict the risk and severity of future outbreaks, and evaluate the efficacy of different surveillance strategies in preventing future epidemics^22–24^. However, network-based modeling approaches are challenging for bTB in part due to the chronic nature of the disease, characterized by long latent periods, low within-farm transmission rates, and limitations of diagnostic tests. The prevalence of bTB within herds is often low and highly variable. This heterogeneity is likely to impact the probability of between-farm transmission. Therefore, to more accurately estimate between-farm spread of bTB, transmission models must run over long time periods and incorporate within-farm dynamics, including changes in within-farm prevalence over time. Few between-farm models exist for bTB, many of which do not account for within-farm dynamics^7^. However, recent integrated within- and between-herd bTB models have been developed for the UK and Italy to assess alternative surveillance strategies for those countries^13, 14^.

Uruguay is a South American country with low bTB prevalence and a comprehensive animal traceability system. Despite considerable investment in a test-and-cull program for the control of bTB, the incidence of the disease has increased since 2008 (~4 farms per year in the early 2000s to ~22 per year in 2012–2014), raising concern among stakeholders and animal health agencies^11^. No wildlife reservoir has been identified within Uruguay, and all detected cases have been in dairy farms. The current surveillance program includes a combination of active surveillance in dairy farms (annual intradermal tuberculin testing; caudal fold test -CFT- with confirmation via the comparative cervical test-CCT) and passive surveillance in all farms (slaughter inspection of carcasses for bTB-like lesions)^11^. Risk-based surveillance has the potential to optimize ongoing control programs in low prevalence countries such as Uruguay, balancing the efficacy of control efforts while minimizing investment of human and financial resources. An empirical assessment of risk factors for bTB, combined with computational network-based models to simulate the relative effectiveness of risk-based control measures, is critical for the development of risk-based surveillance in Uruguay.

The primary motivation of this work is to advance understanding of bTB transmission patterns and effective surveillance strategies for regions with low prevalence, yet persistent infection. To achieve this, we designed a within- and between-herd epidemiological model for bTB in the Uruguayan cattle industry, parameterized with real-world incidence data, that simulates observed epidemiological patterns. This model utilizes data available through Uruguay’s Ministry of Livestock, Agriculture and Fisheries from ~45,000 cattle farms, of which 10% are dairy farms, and 500,000 records on the movement of 18 million cattle available for a 5.5 year period^25^. Using this model, we explore alternative surveillance options for bTB to identify targeted strategies that simultaneously minimize surveillance effort and farm-level bTB incidence.

## Results

**Figure 1:**
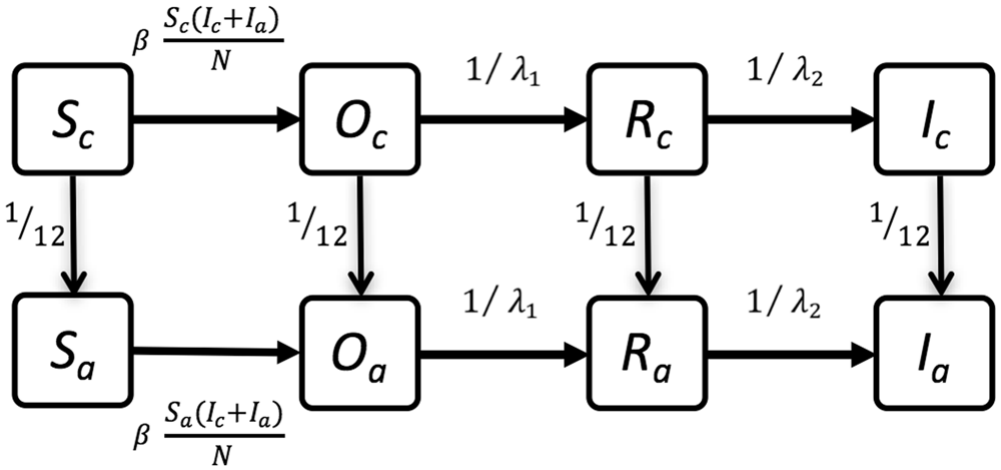
Graphical representation of the compartmental model used to represent within-herd transmission dynamics, including calves (**c**, top row) and adults (**a**, bottom row). Number of individuals in each infection class is indicated as susceptible (*S*), occult/exposed (*O*), reactive (to skin testing, *R*), and infectious (*I*). Total herd size is represented as *N. β* indicates the rate of transmission between infectious and susceptible individuals, and *λ*_*1*_ and *λ*_*2*_ represent the duration of the occult and reactive periods, respectively. Calves transition to adults after twelve months, which is equivalent to 1/12 on the monthly time scale of the model.

We developed an integrated within- and between-farm transmission model to simulate the spread of bTB in Uruguay (see Methods, Supplementary Methods, and Supplementary Table S1). Within-farm transmission was accounted for using an age-structured, stochastic compartmental model (Fig. 1), including infection classes of Susceptible (S), Occult (O: exposed, not reactive to diagnostic tests, not infectious), Reactive (R, not infectious but reactive to skin testing), and Infectious (I, reactive to diagnostic tests and infectious to others). Between-farm transmission occurred via animal movements, which were represented with the observed dynamic movement network over 5.5 years, and via a spatial transmission kernel to capture localized transmission (e.g., through fence line contact, shared equipment, or movement of people that may function as mechanical vectors for fomites). Following the current surveillance program used within Uruguay, the model simulated detection of bTB-positive farms through annual skin testing in all adult dairy cattle and via carcass inspections at slaughter for both dairy and non-dairy animals. Contact tracing and movement restrictions were implemented for detected farms. This model was fit to observed epidemiological data using a genetic algorithm (GA), a machine learning approach for optimizing model parameters within multi-dimensional parameter space^26^, so that model simulations reproduced observed epidemiological dynamics. Observed data used for model fitting included the number of farms detected per year, method of detection (slaughter versus skin testing), and distribution of pairwise distances between infected farms. The fitted model was then used to evaluate the efficacy of alternative surveillance strategies.

### Parameterization and validation

In the model, all dairy animals were tested annually and infected animals (R or I) were detected with probability *sens*_*sk*_, the animal-level sensitivity for skin testing. For slaughter surveillance, infectious animals were detected with probability *sens*_*sl*_. Additional parameters fit by the GA were those that define the transmission kernel; the probability of transmission between farms was assumed to scale linearly with the prevalence in the infected farm and decrease exponentially with distance. The shape of the kernel was controlled by two parameters, *ϕ* and *α*.

**Figure 2:**
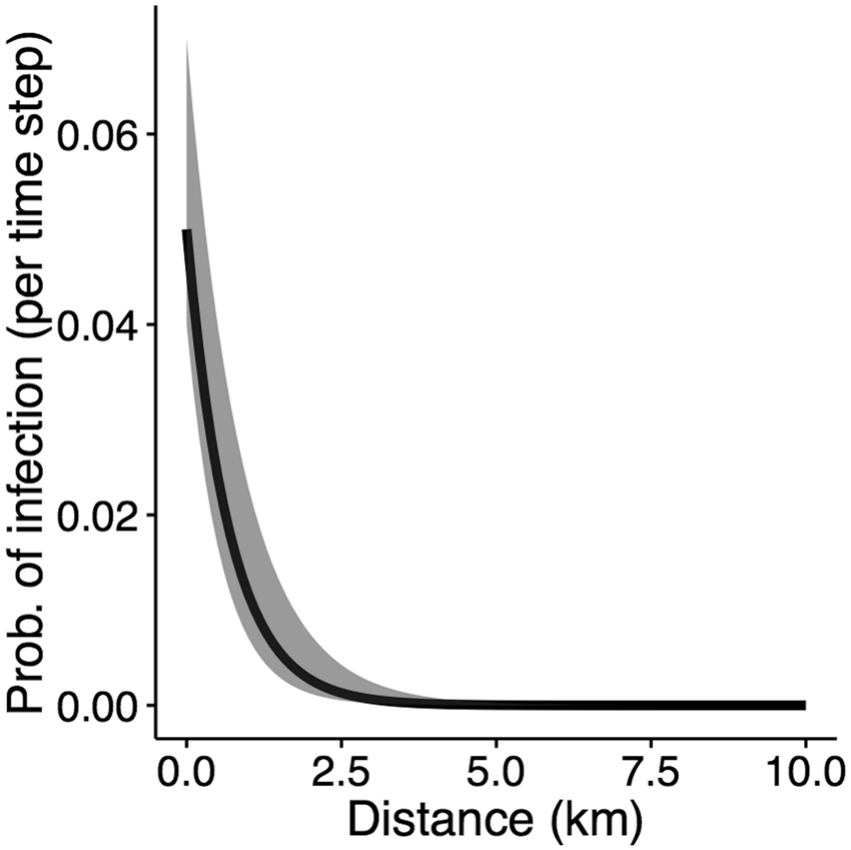
Fitted spatial transmission kernel describing the probability of transmission between two farms by distance when the prevalence in the infected farm is 1. The gray area represents uncertainty in the parameter estimates (Table 1).

**Figure 3:**
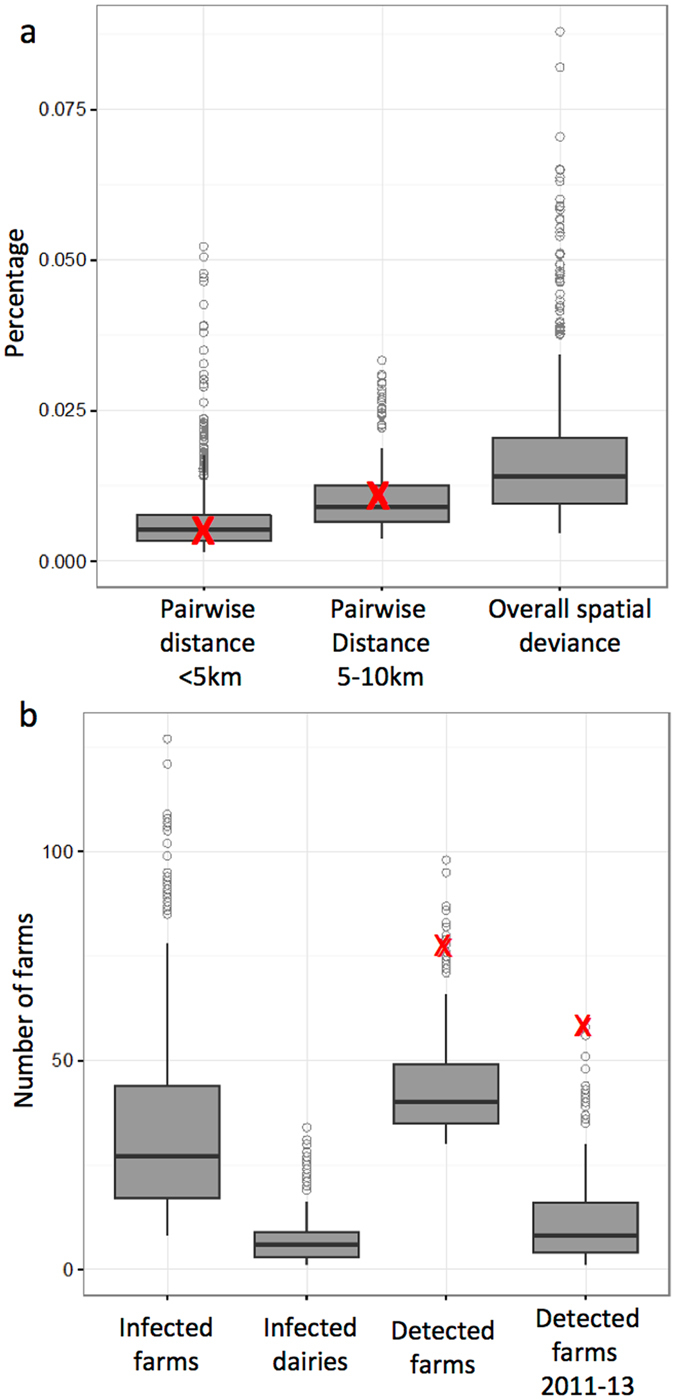
Summary of the epidemiological dynamics of simulations run with the fitted parameters in terms of (**a**) the proportion of pairwise distances between infected farms that are 0–5 and 5–10 km apart, as well as the percent spatial deviance in simulated epidemics, and (**b**) total number of infected farms, infected dairies, detected farms (overall: 2008–2013), and detected farms (2011–2013, the time period used for model parameterization). “X”s represent the observed data. No observed data are shown for infected farms because the true number of infected farms in the population is not observable.

Fitted parameter values estimated by the genetic algorithm are summarized in Table 1. Estimated animal-level sensitivities for surveillance measures included *sens*_*sl*_ = 0.42 [range: 0.39–0.53] for slaughter inspection and *sens*_*sk*_ = 0.53 [0.46–0.62] for skin testing. Parameters controlling the shape of the transmission kernel were $\varphi$ = 0.05 [0.04–0.07] and $\alpha$ = 1.46 [1.12–1.74] (Fig. 2), and the number of initially infected farms was estimated to be 25 dairies [20–29 dairies]. Based on 1000 runs of the fitted model, we summarized model performance in terms of the pairwise distance between infected farms (proportion of pairwise distances <5 km, 5–10, and 10–20 km apart), total number of detected farms, and proportion of farms detected via each method (slaughter surveillance, skin testing, contact tracing). These distributions were compared with real-world data to further assess the model’s fit. The observed values fell within the interquartile range for the spatial criteria and showed low spatial deviance (Fig. 3a), indicating that the observed data were consistent with the epidemiological dynamics predicted by the model. The total number of detected farms in the observed data exceeded the 75^th^ quartile of the simulations, but still fell within the overall range predicted by the model (Fig. 3b, Table S2). In other words, the observed epidemiological data could represent one realization of the epidemiological dynamics represented by the model. In addition, despite being seeded in dairy farms, the model consistently predicted bTB spread to non-dairy farms, accounting for well over half of all farms detected. In contrast, non-dairy infections were not reported in the observed data, although non-dairy farms were not subjected to annual skin testing.

**Table 1:** Definitions, estimated values, and constraints for parameters undergoing optimization.

Parameter	Definition	Optimization constraints (min, max)	Estimated value (range)
*Surveillance* (*at animal-level*)			
*sens*_*sl*_	Sensitivity of slaughter surveillance	(0.3, 0.7)	0.42 (0.39–0.53)
*sens*_*sk*_	Sensitivity of skin test	(0.3, 0.7)	0.53 (0.46–0.62)
*Spatial transmission kernel*			
*Φ*	Spatial transmission coefficient	(0.01, 0.1)	0.05 (0.04–0.07)
*α*	Shape of spatial transmission kernel	(1, 2)	1.46 (1.12–1.74)
*Initial conditions*			
*seeds*	Number of farms infected initially	(10, 30)	25 (20–29)

### Testing alternative surveillance strategies

We evaluated surveillance strategies that eliminated active surveillance in low-risk dairies, while maintaining existing surveillance measures on high-risk dairies (Table 2)^11^. Specifically, lower risk farms that failed to meet cut-off values for herd size and/or number of animals received did not undergo annual skin testing (no active surveillance), whereas high-risk farms that exceeded cut-off values underwent annual skin testing. The relative effectiveness of the baseline scenario and nine alternative surveillance strategies was assessed by comparing the annual incidence of bTB in the final three years of the model period (2011–2013). 1000 simulations were run per scenario.

**Table 2:** Criteria for farms undergoing annual skin testing for 10 surveillance scenarios. Size criteria refers to the herd size of the farm in the current year. Movement criteria refers to the number of animals received in the previous three years.

Scenario	Type	Size criteria	Movement criteria	Description of farms tested annually
Baseline	Dairy	—	—	All dairies
A	Dairy	> 360 &	> 44	High-risk by size AND movements
B	Dairy	> 115 &	> 1	Med/High-risk by size AND movements
C	Dairy	> 360 OR	> 44	High-risk by size OR movements
D	Dairy	> 115 OR	> 1	Med/High-risk by size OR movements
E	Dairy	> 360	—	High-risk by size
F	Dairy	> 115	—	Med/High-risk by size
G	All		Top 10^th^ percentile	High-risk by movement
H	Dairy	—	—	Improved slaughter surveillance (10%)
I	Dairy	> 115 OR	> 1	Scenario D + all dairies biennially

**Figure 4:**
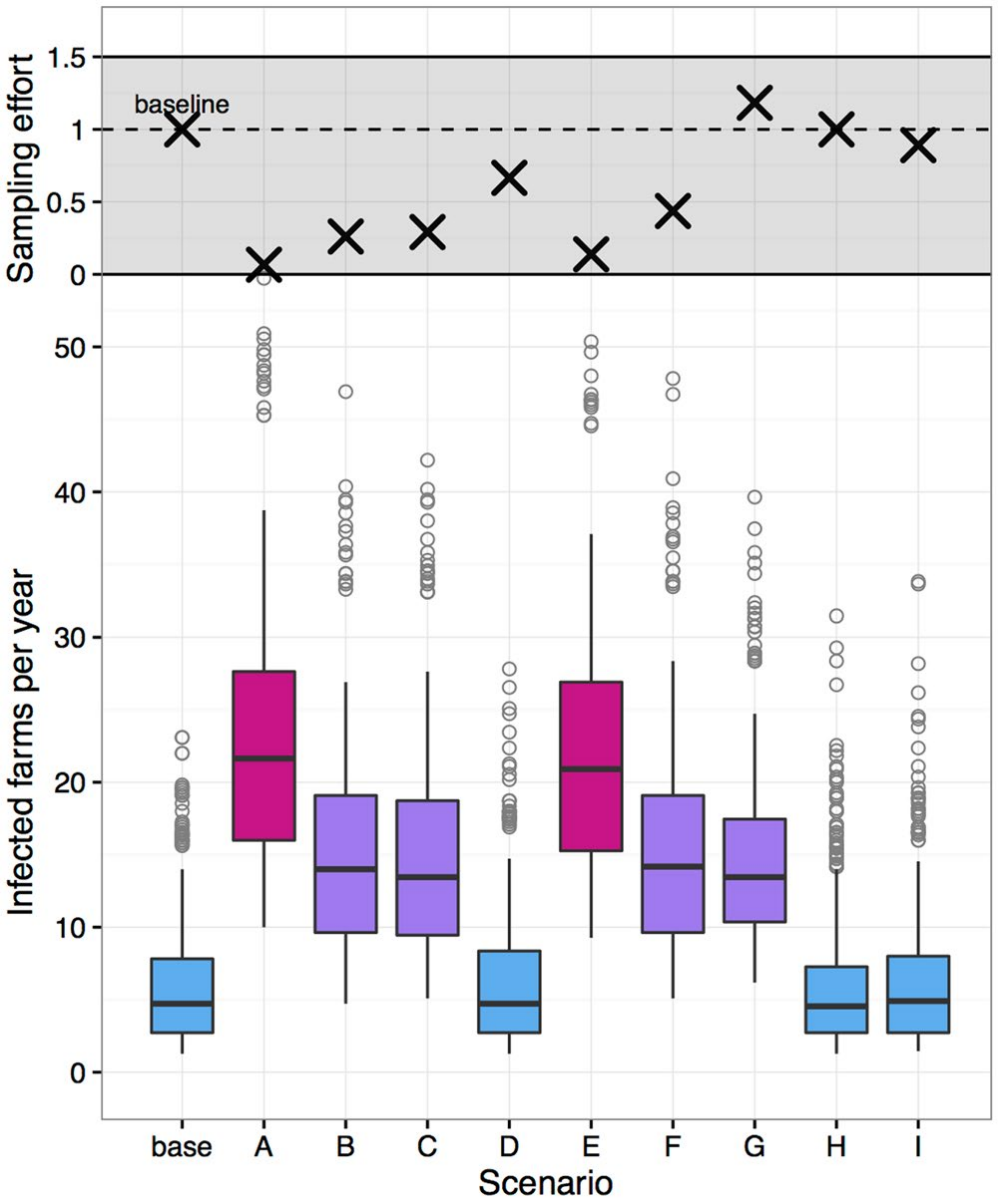
Comparison of sampling effort (top panel) and number of infected farms (bottom panel) for alternative surveillance strategies. Boxplot shows the number of farms infected per year (median, interquartile range, and 95^th^ percentile whiskers), and open circles represent outliers. Scenarios depicted with the same color were not significantly different based on a Kruskal-Wallis test. Sampling effort is calculated as the number of farms tested per year relative to the baseline scenario (~4,400 farms).

We identified three alternative surveillance strategies (Scenarios D, H, and I) in which the number of infected farms per year did not differ significantly from the baseline scenario (Fig. 4). Of these, Scenario H and I required similar numbers of farms to be tested annually as compared to the baseline, whereas Scenario D reduced sampling effort by 40%. Scenario A (testing farms with herd size >360 that also received >44 animals) and E (herd size >360 head and no movement criteria) resulted in the largest annual incidence, with >20 new infected farms per year, and reduced sampling effort to <25% of the baseline scenario. Scenarios B, C, F, and G resulted in annual incidence significantly lower that A and E but also significantly higher than baseline. Scenarios B, C, and F required testing 25–50% fewer farms than the baseline scenario, and Scenario G increased the number of farms sampled (risk-based surveillance based on movements only, ignoring production type and herd size). Notably, D and F highlight the importance of including movement criteria in defining which farms to test. Scenario F only uses a size-based threshold (>115 herd size), whereas D uses the same size-based threshold in combination with a movement threshold (farms must receive at least 1 animal in the previous three years).

### Model sensitivity analysis

We found that the epidemiological dynamics predicted by the model were sensitive to all parameters explored $(\varphi$, $\alpha$, *sens*_*sl*_, *sens*_*sk*_), as measured by the total number of infected and detected farms and the percentage of pairwise distances between infected farms that were <5 km (Table 3). As expected, increasing the sensitivity of surveillance measures resulted in smaller numbers of farms infected, and thus, subsequently detected. Increasing $\varphi$ increased the likelihood of local (<5 km) transmission between farms and increased the total number of farms affected, whereas increasing $\alpha$ (i.e., increasing the steepness in which transmission probabilities decline with distance) had the opposite effect. While these results are intuitive, they serve as verification that the model is behaving in a manner consistent with epidemiologic expectations^27^.

**Table 3:** Results of sensitivity analysis. PRCC (Partial rank correlation coefficients) between model parameters and key model outputs. *Indicates significant PRCCs (p < 0.05).

Model output	Parameter			
	*Sens*_*sl*_	*Sens*_*sk*_	*ϕ*	*α*
Total infected farms	−0.46*	−0.76*	0.34*	−0.37*
% infected farms that were < 5 km	−0.13	−0.25*	0.65*	−0.60*
Total number of farms detected	−0.47*	−0.80*	0.25*	−0.26*

## Discussion

The goal of our study was to model the spread of bTB and assess the efficacy of current and alternative surveillance strategies for controlling this pathogen in low prevalence settings, such as Uruguay. While Uruguay’s test-and-cull control program has been successful at reducing bTB to its current low prevalence (<0.05% of farms infected), programs based on routine testing may not be the optimal strategy for limiting bTB transmission in low prevalence settings. Given the availability of animal movement data, targeted surveillance strategies may both minimize surveillance costs and improve control. To explore the potential for targeted surveillance within this low prevalence country, we designed a network-based transmission model that integrated within- and between-farm transmission processes and optimized parameter values with real-world data to emulate the epidemiological dynamics specific to Uruguay. This model was used to test alternative surveillance strategies.

We focused on alternative surveillance scenarios that reduce the number of farms tested by eliminating annual testing in low-risk dairy farms. The analysis presented here identified one scenario in particular, Scenario D, in which annual testing was reduced by 40% without increasing incidence. Scenario D eliminated testing on low-risk farms, which were defined as dairies with fewer than 115 head and that had purchased one or fewer animals over the preceding three years. Annual testing was retained on farms with higher risk, as defined by exceeding the minimum threshold for either herd size or movements. The best scenario was likely effective because between-farm transmission chains were likely to include at least some high-risk farms that would be detected as part of annual skin testing, and low-risk farms would be detected as part of contact tracing. Testing low-risk dairy farms every other year (Scenario I) did not further reduce incidence, likely because these farms would be detected through contact tracing regardless of biennial testing. While the incidence of bTB was not reduced below baseline levels in Scenario D, this scenario is a promising option given substantial reductions in testing effort.

Scenarios A, B, C, and E resulted in higher bTB incidence than the baseline or Scenario D, likely because annual testing was eliminated on a greater proportion of dairy farms as a result of more restrictive thresholds for herd size and movements. It is also worth noting that Scenario F performed more poorly than D; scenario F used the same herd size criterion as D but ignored movement data, thus highlighting the importance of the availability and use of movement data in improving targeted control efforts. Similarly, ignoring herd size data and targeting control efforts based on movement alone (including dairies and non-dairies, Scenario G) did not reduce incidence beyond the baseline. Improving slaughter surveillance (Scenario H) by 10% also had limited impact on predicted incidence, suggesting that any training or reward programs meant to increase the effectiveness of slaughter surveillance would need to increase the sensitivity of slaughter surveillance by more than 10% to expect any substantial impact on bTB incidence. Future work could explore additional scenarios that depart more substantially from current surveillance measures, such as pre-movement testing, targeting of high-risk non-dairy farms, and the use of more sensitive diagnostics in high-risk farms, such as the interferon gamma assay. Additionally, we primarily focused on risk in relation to animal movements and thus far have not explored targeted measures based on spatial variation in risk, such as farm density or spatial clusters of bTB-positive farms^11^. We plan to evaluate additional surveillance strategies based on spatial definitions of high-risk farms and alternative testing procedures in the future.

Although movements and farm size are dynamic through time, targeted surveillance based on these criteria is feasible in Uruguay. High-risk farms were defined based on movement in the previous years, not the year of testing. Given that the movement database is actively maintained by the Uruguayan government, it would be possible to extract movement data from the previous three years in order to define the present year’s high-risk farms. Furthermore, both a farm’s tendency to engage in movements and its herd size are correlated across years^25^. Thus, implementation of a targeted strategy is viable and economical. Given that the cost of annual testing is borne by the farmer, a low-risk farm with a median size herd of ~100 head would save approximately $450 USD annually, which is sizeable considering Uruguay’s GDP per capita is ~$15,500^28^.

The effectiveness of targeted skin testing demonstrated by our model deviates from modeling conclusions in the United Kingdom, where targeted surveillance based on numbers of outward movements from farms did not substantially alter the effectiveness of control programs^13^. However, their focus on outward rather than incoming movements to farms may not as effectively target farms at high-risk of becoming infected. In addition, the UK differs markedly from the situation in Uruguay in that herd-level prevalence is substantially higher (9–12% in England and Wales) and a wildlife reservoir is present in Europe^13, 29^. In bTB-free regions of Italy, models predict that slaughter inspection and routine on-farm testing are more crucial to surveillance than pre-movement testing, which is a measure currently not part of Uruguay’s control program^14^. However, targeted surveillance options are often not considered in bTB-free settings^14, 30^.

To our knowledge, this work represents the first use of genetic algorithms for parameterizing epidemiological models in veterinary epidemiology. We suggest that GAs are a promising tool for parameterizing complex epidemiological models, particularly when trying to fit a model to several types of observed data (e.g., spatial distribution and case counts). Parameter estimates for the transmission kernel represent one of the only estimates existing in the literature for estimating localized transmission processes between farms. For example, the UK bTB model handles local transmission by considering all farms located within the same parish to be equally at risk^13^, and unlike Uruguay, the transmission kernel is driven by a wildlife reservoir. Thus, our estimates will likely be useful in parameterizing bTB models for settings with little or no wildlife contribution to spread.

The sensitivity of serially applied skin tests (CFT-CCT) is estimated to range from 74–93%^31^. However, test sensitivity under field conditions is often far lower^32, 33^, and our estimate of ~50% falls within the range reported elsewhere for serial testing under field conditions^32–34^. Similarly, the sensitivity of slaughter inspection was estimated to be 42% for infectious animals and 21% for reactive animals, which is consistent with values reported elsewhere^35–38^. Importantly, our estimates of sensitivity include the entire diagnostic process, and thus a false negative may occur for reasons beyond poor performance of the diagnostic tests in itself (e.g., inadequate cold chain for samples of bTB-like lesions detected at slaughter). Our sensitivity estimates did not distinguish among multiple possible reasons for which infected animals are not reported. Additional training in either the application of skin tests or recognition of bTB-like lesions at slaughter could improve the sensitivity of surveillance in the country.

While the spatial fit of our model was adequate, the model underestimated the number of new infections per year. This could be due to our assumption that control was perfect, whereby there were no undocumented movements from infected farms under movement restrictions and culling of infected animals was immediate. In reality, farms often remain infected for more than a year after the imposition of control measures and prior to clearing infection. These farms could potentially be a source of infection to neighbors. We also assumed that, for slaughter surveillance, the probability of detecting cattle in the reactive stage of infection was half that of infectious individuals^14^. If the sensitivity of detecting reactive cattle is, in practice, far lower than was assumed, this would delay the detection of infected farms and potentially allow for more extensive between-farm spread prior to detection. In addition, we only allowed for between-farm, and not inter-individual variation in the sensitivities of skin testing and slaughter surveillance, which may have increased the probability of detecting infected farms. These factors relating to bTB control may have led the model to overestimate the effectiveness of control measures, and thus prematurely halt transmission chains as compared to the real situation in Uruguay. While absolute numbers of cases may be underestimated, we believe that our results on the relative effectiveness of alternative surveillance scenarios are robust.

Although the model was always seeded in dairy farms, the majority of the cases were in non-dairy farms (Fig. 3b), demonstrating substantial opportunity for dairy to non-dairy transmission via observed patterns of animal movements. In contrast, 57 of 58 bTB-positive farms detected in Uruguay between 2011 and 2013 were dairy farms, all of which were detected via skin testing. The model predicted that the majority (85%) of detections in dairies involved skin testing, which corresponds reasonably with the observed data (Supplementary Table S2). In addition, a larger proportion of the infected dairies were detected, whereas non-dairies were more likely to remain undetected (Supplementary Table S2). Detections of non-dairies in the model were generally split equally between detection at slaughter and via contact tracing. Non-dairy farms do not undergo skin testing, making it difficult to ascertain whether the model’s predictions on bTB in non-dairy farms is erroneous or if the observed data underestimates the true prevalence in non-dairies due to lack of active surveillance. The results from our model suggest that future research should investigate the prevalence of bTB in the beef sector of Uruguay’s cattle industry.

Underlying risk factors related to the transmission of bTB create heterogeneities in infection patterns that can be used to target surveillance and control efforts. We explored optimal strategies for reducing the sampling effort required to maintain low bTB prevalence, and identified specific strategies for targeted surveillance based on a combination of movement and herd size criteria that reduce sampling effort by 40% relative to the surveillance program currently employed. The surveillance scenarios identified were sufficient to prevent an increase in the prevalence in bTB while minimizing sampling effort, but did not achieve a reduction in prevalence relative to the current surveillance program. Our exploration of alternative strategies was not exhaustive, and future directions include a more thorough investigation of the targeted use of alternative diagnostic tests and testing in dairy and non-dairy herds, with the aim of identifying strategies that move the country from maintenance of low bTB prevalence to bTB eradication. An economic analysis would also be beneficial to more thoroughly assess the costs and benefits of each strategy. Conclusions and lessons learnt about the relative efficacy of targeted surveillance strategies in Uruguay are applicable to other countries or regions with low bTB prevalence.

## Methods

### Data source

Data on farm attributes and between-farm cattle movement from July 2008 to May 2013 were obtained from the Uruguay’s Ministry of Livestock, Agriculture, and Fisheries^25^, including the geographic location as UTM coordinates (Universal Transverse Mercator), herd size, and production type of each farm. Mean number of premises recorded per year was ~45,000, with dairies accounting for ~10% of all farms. Movement records consisted of the date of each movement, total number of animals of each age-class moved (calves <12 mo.; adults >12 mo.), and the premise ID of the source and destination farms. A full characterization of the cattle herd demographic and movement data is described in VanderWaal *et al*.^25^, and a detailed summary is included in the Supplementary Methods.

### Model description

We developed an integrated within- and between-farm model to simulate the spread of bTB. This stochastic model tracked transmission processes both within infected farms and allowed transmission to occur between farms by either movement of infected animals or through a local spatial transmission kernel that accounts for localized processes that could contribute to transmission, such as fence line contact, contaminated sewage or water, undocumented local movements of animals (e.g., sharing of bulls or escaped animals), and shared equipment/personnel that may function as mechanical vectors for fomites^39–42^. The model operated on a monthly time step for 5.5 years, which was the period in which data on movements were available.

Within-farm transmission dynamics were captured with an age-structured Susceptible-Occult-Reactive-Infectious (SORI) compartmental model with homogenous, frequency-dependent transmission (Fig. 1)^32, 43^. Infectious individuals (I) infect susceptible individuals (S) at rate *β*, after which the susceptible animal moves into the occult stage (O: exposed, not reactive to diagnostic tests, not infectious). After the occult period (*λ*_*1*_), infected individuals progress into the reactive compartment (R, not infectious but reactive to skin testing). After the reactive period (*λ*_*2*_), animals progress into the infectious stage (I), where they are both reactive to diagnostic tests and contribute to new infections (Fig. 1). Calves transition to adults at the rate of 1/12 (i.e., after 12 months). Susceptible and occult calves are likely to transition to adults prior to reaching the reactive or infectious calf classes, hence reactive and infectious animals are usually adults. This model includes births and slaughter. Slaughter rates and transmission coefficients *β* were specific to dairy and non-dairy production types^44, 45^. A full description of the stochastic within-herd transmission model and model parameters can be found in the Supplementary Methods (Supplementary Table S1).

### Between-farm transmission via animal movements

For each time step *t*, all movements involving infected farms at time *t* were extracted from the movement database. The total number (batch size *b*) of adults or calves moved in the model was drawn from a Poisson distribution centered on the observed batch size for the specific age-class in that particular movement in the movement database. For each compartment in Fig. 1, the number of animals moved was determined by drawing from Binom (*b*, $\frac{{n}_{s}}{n}$), where *n*_*s*_ is the total number of animals in a given age-class and infection stage, and *n* is the total number of animals in that age-class. The numbers of animals in each class were updated on the origin and destination farms. If no infected animals remain on the origin farm after movement, then that farm is considered to have cleared the infection and is re-classified as uninfected.

### Between-farm transmission via local spread

Local spread occurs via a spatial transmission kernel, where the probability of transmission decreases as two farms become farther apart:

$$P(far{m}_{j}becomes\,infected)=1-\,\prod _{1}^{i}(1-\,\frac{{I}_{i}}{{N}_{i}}\varphi {e}^{-\alpha {d}_{ij}})$$

Where farm *j* is an uninfected farm and *d*_*ij*_ is the distance between farm *j* and every infected farm *i* within 20 km. Distances of >20 km were not considered as local spread at long distances is unlikely^46^. $\frac{{I}_{i}}{{N}_{i}}\varphi {e}^{-\alpha {d}_{ij}}$ represents the probability of transmission between farm *i* and *j* (scaled by infection prevalence in farm $i,\frac{{I}_{i}}{{N}_{i}}$) given distance apart in kilometers, and thus 1−$\frac{{I}_{i}}{{N}_{i}}\varphi {e}^{-\alpha {d}_{ij}}$ is the probability that transmission will not occur between those two farms. *ϕ* and *α* control the shape of the transmission kernel, with *ϕ* indicating the probability of transmission when *d*_*ij*_ = 0, and *α* controlling the steepness with which probabilities decline with distance. If a farm becomes infected via local spread during a time step, one susceptible adult is reclassified to the occult stage.

### Surveillance and control measures

Following Uruguay’s existing surveillance and control program^11^, all animals sent to slaughter undergo passive surveillance via carcass inspection. Each infectious animal can be detected in the slaughterhouse with probability *sens*_*sl*_, and following Rossi *et al*.^14^, we assume that animals in an earlier stage of infection (i.e., reactive animals) are detected at half this probability because they are expected to be in a less apparent stage of infection (see Supplementary Methods)^14, 47^. In addition, all dairy animals > 1 year old undergo active surveillance in Uruguay, which involves annual skin testing with the caudal fold test (CFT) and confirmation of reactors using the comparative cervical test (CCT). Each reactive and infectious animal is detected with probability *sens*_*sk*_, which is defined as the combined sensitivity to the CFT-CCT. To account for variation in the performance of surveillance activities, values for *sens*_*sl*_ and *sens*_*sk*_ are drawn from a beta distribution each time they are performed on a batch of animals at a farm, with mean equals to *sens*_*sk*_ or *sens*_*sl*_ and variance equal to 0.012 (Supplementary Table S1)^13^.

Any farm with at least one detected animal is reclassified as a detected farm and control measures based on the current test-and-cull control program utilized within Uruguay are implemented. All movements from the detected farm are restricted. For simplicity, we consider control measures to be perfect in that no illegal movements occur and that the farm also no longer contributes to local spatial spread. Detected farms are permanently removed from the simulation and do not re-enter the population of susceptible farms. This is a reasonable assumption, given that of the 58 detected farms occurring in Uruguay between 2011 and 2013, only 13 of the farms had been certified clear of bTB by the end of 2013.

In addition to control measures on detected farms, contact tracing occurs for all of the farm’s connections in the movement network for a period of two years prior to the detection date. Geographic neighbors are also identified. Neighbors and contacts undergo skin testing as described above, and any farms that are detected undergo the same control measures.

### Model calibration

We conducted a multivariate calibration exercise on parameters directly involved with between-farm transmission and surveillance ($\varphi$, $\alpha$, *sens*_*sl*_, *sens*_*sk*_) using Latin Hypercube Sampling (LHS) and partial rank correlation coefficient (PRCC) analyses. This approach has often been used for global sensitivity analyses in disease models and agent-based models^48–51^. We generated 500 parameter sets through sampling a Latin Hypercube, which is expected to efficiently cover the parameter space (see Table 1 for minimum and maximum values for each parameter). Because bTB is endemic in Uruguay and 57 of 58 infected farms were dairies^11^, model runs were seeded in ten randomly selected dairy farms. Initial conditions are further described in the Supplementary Methods.

One hundred simulations were conducted per parameter set, yielding 50,000 simulations. Each simulation set ran on the same 100 sets of index cases to eliminate variability associated with index case choice. PRCC analysis was based on the averaged values from the simulation sets^52^. Specifically, we used Spearman’s ranked partial correlation coefficients to detect monotonic relationships between model parameters and outputs after accounting for the effects of all other parameters^53^. Outputs included the total number of infected farms to assess overall model performance, the total number of farms detected during 2011–2013 to assess sensitivity to bTB diagnostic parameters, and the percentage of infected farms that were < 5 km apart to assess sensitivity to the spatial transmission kernel parameters.

The results of the LHS analysis indicated that only 75 simulations per parameter set were necessary to obtain a consistent result, as described in the Supplementary Methods^52, 54, 55^. Based on this analysis, 75 simulations per parameter set were used for subsequent model parameterization.

### Model parameterization and validation

A genetic algorithm (GA) was used to optimize parameter values in the model so that simulations matched observed epidemiological dynamics^26^. GAs are a class of machine learning methods for model optimization that are used to find optimal solutions in multidimensional parameter space. GAs are based on the mechanics of biological evolution and aim to find parameter values that maximize the “fitness” of the model, where fitness is a user-defined function that quantifies how well model outputs match observed data^26^. To do this, the GA first generates a population of parameter sets, running 75 simulations per set and calculating the average fitness of simulations within each set. Based on the principles of natural selection, parameter sets that produced high fitness simulations are “selected” to propagate into the next generation of parameter sets, with some degree of mutation and crossover. This process is repeated for many generations in order to optimize parameter values^26^.

The fitness function was defined based on three components (*k*) that quantify the deviance between the observed and simulated epidemic in terms of spatial dynamics, total number of farms detected, and detection method. The spatial deviance component was calculated by first measuring the pairwise distances between all infected farms, and summarizing the distribution of pairwise distances by calculating the proportion of distances that fell < 5 km, 5–10 km, and 10–20 km apart. The spatial deviance function was defined as the summed absolute differences between the observed and predicted proportions, where high values indicated a greater deviance from observed values. Deviance in the total number of detected farms (farm deviance component) was calculated as the percent difference between the observed and predicted number of detected farms in the last three years for a given parameter set. For the predicted number of detected farms, the 75^th^ quantile of model runs was used instead of the average because bTB went extinct within the first year in a large proportion of simulations. Deviance in detection method (detection method deviance component) was calculated as the summed difference in the observed and simulated proportion of detected farms that were detected via skin testing, slaughter surveillance, or contact tracing. Deviances of each component was re-scaled by dividing by the maximum deviance observed in all LHS runs in order to ensure that all components exhibited similar magnitudes in their values. A variable-weighting strategy, in which each component received a different weight each time fitness was calculated, was employed to ensure that any one component cannot dominate the optimization process^26, 56–58^. The overall fitness function is then:

$$\,f(x)\Rightarrow \mathop{{\rm{\max }}}\limits_{x}(-\sum _{i=1}^{k}{w}_{i}{f}_{i}(x))$$

Where *f*_*i*_(x) is the function for calculating each deviance component, and {*w*_*i*_} is a set of positive values used for weighting whose elements sum to one. {*w*_*i*_} is randomly generated each time fitness is calculated.

Because there are multiple operators that determine the exact mechanics of how selection, mutation, and crossover occur, we tuned the GA by simulating an epidemic with known parameter values and then identifying operators for selection, mutation, and crossover that were able to recover the known parameter values to within 10%. See the Supplementary Methods for details on tuning the genetic algorithm.

We applied the tuned GA to the real-world data to estimate parameter values. 1000 simulations were then performed with the GA-fitted parameter values to validate model performance. Distributions of the pairwise distance between infected farms, total number of detected farms (2011–2013), and detection methods were extracted from simulated outputs and compared with the real-world data to assess model fit. After the initial optimization, the model consistently underestimated the number of observed outbreaks. Thus, we re-ran the GA holding the spatial parameters at the previously fitted values and allowed the GA to fit the number of farms initially infected (seeds) concurrently with *sens*_*sl*_ and *sens*_*sk*_. All GAs were run with the *GA* package in R v3.2.3^59^.

### Testing alternative surveillance strategies

Dairy farms were classified as low or high-risk for bTB infection based on an epidemiological analysis of bTB-positive dairy farms in Uruguay^11^. Risk classes were defined using a combination of criteria based on the number of animals received by a farm in the previous three years and the herd size of the farm. We explored the efficacy of surveillance strategies that reduced active surveillance in lower-risk farms (defined based on criteria related to farm size and movement frequencies), while maintaining existing levels of surveillance on high-risk farms (Table 2). Specifically, farms that failed to meet cut-off values for herd size and/or number of animals received did not undergo annual skin testing (no active surveillance), whereas farms exceeding cut-off values for herd size and/or number of animals received underwent annual skin testing. Table 2 summarizes criteria for farms receiving annual testing for each scenario. Cut-off values described in Table 2 for herd size and movement criteria were based on results of a bTB case-control study in Uruguayan dairy herds, which showed that herds meeting the criteria in Table 2 were more likely to be infected with bTB^11^. We also tested a scenario in which *sens*_*sl*_ was increased by 10%. 1000 simulations were run per scenario. The relative effectiveness of alternative strategies was assessed by running the model for 2008 to 2013 and then comparing the annual incidence of bTB in the final three years of the model period (2011–2013), which is the timeframe for which observed epidemiological data were available. Differences in incidence were compared using the Kruskal-Wallis test^60^.

### Data availability

The datasets generated and/or analyzed during this study are not publically available because the data belong to the government of Uruguay and contain confidential information about privately owned farms. However, model outputs are available from the corresponding author on reasonable request.

## Electronic supplementary material

Supplementary material
