# Supplementary material for: Optimal surveillance strategies for bovine tuberculosis in a low-prevalence country
Source: Sci Rep. 2017 Jun 23;7:4140. doi: 10.1038/s41598-017-04466-2 (PMC5482878; doi:10.1038/s41598-017-04466-2)
Supplement: Supplementary file 1 — Supplementary material [file 41598_2017_4466_MOESM1_ESM.pdf]

SUPPLEMENTARY MATERIAL:
Optimal surveillance strategies for bovine tuberculosis in a low-prevalence country

Kimberly VanderWaal, Eva A. Enns, Catalina Picasso, Julio Alvarez, Andres Perez,
Federico Fernandez, Andres Gil, Meggan Craft, Scott Wells

Table of Contents:

Supplementary Methods

1. Table S1: Parameter definitions and distributions
2. Summary of cattle herd demographic and movement data
3. Within-herd model description
4. Initial conditions
5. Evaluation of number of simulations per parameter set
6. Genetic algorithm tuning

Supplementary Results

1. Table S2: Observed epidemiological data and corresponding predictions from 1000 runs of fitted model

References

Supplementary Methods

Supplementary Table S1: Parameter definitions and distributions

Parameter	Definition	Distribution	Units	Citations
**Within-herd dynamics**				
β	Transmission coefficient – dairy	log-normal (mean = 0.39, sd = 0.28)	new cases/ case/month	1
β	Transmission coefficient - non-dairy	log-normal (mean = 0.23, sd = 0.15)	new cases/ case/month	1
λ_1	Duration of Occult period	PERT(0.47, 3.33, 17.23)	months	2-5
λ_2	Duration of Reactive period	PERT(3, 6, 36)	months	2-4,6-8
**Slaughter rate for production type *p*:**		Poisson		9
μ_p	Adult-Dairy	0.27		
	Adult-Breeding	0.17		
	Adult-Complete cycle	0.23		
	Adult-Fattening	0.33		
	Adult-Small farm	0.21		
	Adult-Growing	0.09		
	Calves-all types	0.01		
**Surveillance**				
$Sens_{sl}$	Sensitivity of slaughter surveillance	Beta(mean= *sens.sl*; var = 0.012)	per animal	6-8,10,11
$Sens_{sk}$	Sensitivity of skin test	Beta(mean=*sens.sk*; var = 0.012)	per animal	5,8,10-12
**Initial conditions**				
seeds	Number of farms infected initially		farm	
**Spatial transmission kernel**				
Φ	Spatial transmission coefficient		per month	
α	Shape of spatial transmission kernel			

2. Summary of cattle herd demographic and movement data

Data on farm attributes and between-farm cattle movement from July 2008 to May 2013 were obtained from the Uruguay's Ministry of Livestock, Agriculture, and Fisheries [13]. Farm attribute data consisted of the geographic location as UTM coordinates (Universal Transverse Mercator), herd size, and production type of each farm. Herd size was defined as the reported total number of cattle of any age reported on the farm. Production types were classified into two broad groups: dairy and non-dairy. Dairies accounted for ~10% of all farms. Non-dairy farms were further subdivided into breeding (~42% of farms), fattening (22%), growing (heifers and calves; 2%), complete cycle (15%), and small farms (herd size < 5 cattle; 9%). 62,767 unique livestock premises were recorded during the study period, though not all were in existence for all six years. The average number of premises recorded per year was ~45,000. As with many national level datasets, it is not certain whether the GPS coordinates associated with farms are located at the center of the farm (including pastures) or, more likely, at the farm's main facilities (i.e., gate, barns, etc.). Thus, distance between farms was based on reported GPS locations, which most likely represent distance between the farms' main facilities. However, given the national scale of our model, we do not believe that this source of variation will have a substantial impact on our model outputs.

Movement records consisted of the date of each movement, total number of cattle of each age-class moved, the premise ID of the source location, and the premise ID of the destination. Available records spanned 5.5 years, from July 2008 to December 2013, and included data on the movement of ~3.3 million head annually (~90,000 batches of animals moved annually). Movements for which the final destination was not a farm (i.e., cattle moved to slaughter or exported) were not recorded in the movement database. Changes of ownership involving markets, auctions, or dealers were represented in these data as the seller and buyer with no intermediate location due to lack of information on movements through markets/auctions. Due to the low transmission rate of bTB, mixing of animals at markets was not likely to result in transmission [12]. While non-reported movements of animals may occur with very low frequency, we do not expect such movements to have an impact on this description of cattle movement networks, given the large sample sizes described in this study (>60,000 farms over four years) and that all cattle must be registered in the first six months of life. A full characterization of the cattle herd demographic and movement data is provided in [13].

3. Within-herd model description

Within-farm transmission dynamics were captured with an age-structured, stochastic Susceptible-Occult-Reactive-Infectious (SORI) compartmental model with homogenous, frequency-dependent transmission [1,12]. Homogeneous mixing between calves and adults was assumed. In this compartmental model, infected animals progress through several disease stages, and every individual within the farm's population is classified into one of these compartments (Figure 1). Susceptible individuals (S) have not yet been infected. Following adequate contact with an infectious individual, a susceptible individual moves into the occult stage (O). These animals are exposed, but they do not yet contribute to new infections nor are they reactive to skin testing. After the occult period (duration λ_1), infected individuals progress into the reactive compartment (R), in which they are still not yet infectious, but have the potential to be detected by diagnostic tests. After the reactive period (duration λ_2), animals progress into the infectious stage (I), where they are both reactive to diagnostic tests and contribute to new infections. bTB is a chronic disease for which there is no recovery. Calves (c) are defined as individuals less than one year old, and calves in the S, O, R, and I compartments transition into the corresponding adult (a) compartment after one year. The deterministic skeleton of the transmission model, without age transitions, can be written as the following system of differential equations:

$$\frac{dS_c}{dt} = -\left(\beta\ \frac{S_c(I_c+I_a)}{N}\right); \qquad \frac{dS_a}{dt} = -\left(\beta\ \frac{S_a(I_c+I_a)}{N}\right)$$

$$\frac{dO_c}{dt} = \left(\beta\ \frac{S_c(I_c+I_a)}{N}\right) - \left(O_c\frac{1}{\lambda_1}\right); \qquad \frac{dO_a}{dt} = \left(\beta\ \frac{S_a(I_c+I_a)}{N}\right) - \left(O_a\frac{1}{\lambda_1}\right)$$

$$\frac{dR_c}{dt} = \left(O_c\frac{1}{\lambda_1}\right) - \left(R_c\frac{1}{\lambda_1}\right); \qquad \frac{dR_a}{dt} = \left(O_a\frac{1}{\lambda_1}\right) - \left(R_a\frac{1}{\lambda_1}\right)$$

$$\frac{dI_c}{dt} = \left(R_c\frac{1}{\lambda_1}\right); \qquad \frac{dI_a}{dt} = \left(R_a\frac{1}{\lambda_1}\right)$$

where β is the transmission coefficient, and S, O, R, and I represent the total number of individuals within each class. Following the tau-leap method proposed by Gillespie [14] for incorporating stochasticity at each time step [15], each segment within the above equations is pulled from a Poisson distribution. For example, the number of adults progressing from the occult to reactive class is drawn from a Poisson distribution with mean $\left(O_a\frac{1}{\lambda_1}\right)$. To account for variability in the transmission coefficient based on management and potential strain differences, each farm's β was drawn from a PERT distribution of values found in the literature (Table S1, Figure S1). The within-herd model is embedded and runs concurrently within the between-farm model.

Figure S1. Transmission coefficient distributions for dairy, non-dairy, and overall.

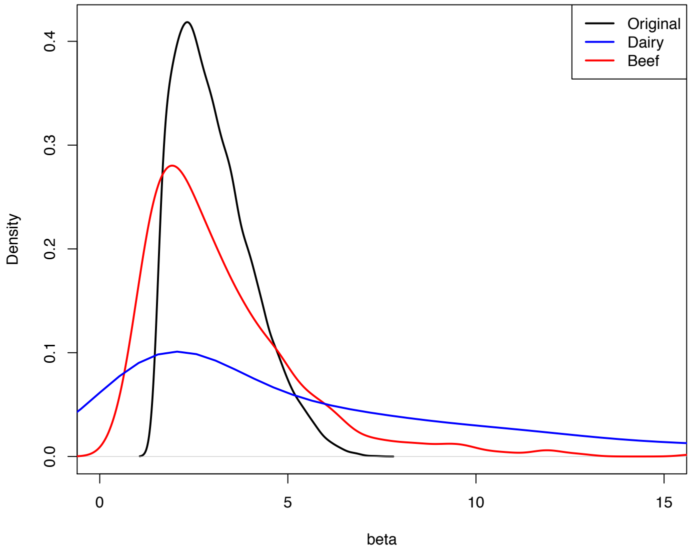

Demographic processes in the within-herd model

In Uruguay, farm size is highly conserved across years (Pearson's correlation coefficient = 0.94)[13]. Thus, births and deaths within a farm were modeled by setting a carrying capacity for each farm, which was equal to the reported farm size for that year. The number of adults and calves slaughtered from each farm was drawn from a Poisson distribution with mean based on the production type's typical slaughter rate (Table S1). Slaughter rates were estimated from published data [9]. Some large farms, usually fattening farms with an abundance of steers, expanded too rapidly to maintain a constant farm size due to large quantities of inward movements of animals. Farms with >500 animals that exceeded their carrying capacity by a factor of 2 slaughtered an additional 25% animals, which served to maintain their herd size to relatively constant numbers. This was a reasonable approximation of variation in herd management in Uruguay, given that a small minority of fattening farms may function as feedlots and thus exhibit unusually high turnover of animals bound for slaughter.

Similarly to the selection of animals for movement, animals to be slaughtered were randomly drawn from each compartment based on a binomial distribution. If no infected animals remain on the farm (either through movements or slaughter), the farm is considered to have cleared the infection without being detected. Animals classified as state I (infectious) were detected at slaughter with probability $sens_{sl}$. One would expect that infectious cattle that have already progressed through the O and R phases of infection would be more likely to have more developed TB-like lesions that would be easier to detect at slaughterhouse inspection compared to TB-infected cattle in earlier phases of infection. Consequently, sensitivity of the macroscopic diagnosis at slaughterhouse inspection would be reduced in animals that have not yet evolved from the R to the I phase. Although this decrease in sensitivity is expected to be variable and dependent on a number of factors, such as age of the infected animals or experience of the staff in charge of the diagnosis, the exact value is difficult to determine in the field given to the uncertainties in TB diagnostics. We have assumed the same value as others [6]. Thus, our model has the assumption that the sensitivity of slaughterhouse surveillance is reduced by a factor of 0.5 in R animals relative to I animals [6].

Births occurred when a farm's size dropped below carrying capacity (due to removal of animals via slaughter or movement). In such cases, new animals were added to the susceptible calf compartment so that the farm size equaled the carrying capacity. Slaughter and birth processes occurred every four months.

4. Initial conditions of model runs

Because bTB is endemic in Uruguay and 57 of 58 infected farms were dairies [16], model runs were seeded in ten randomly selected dairy farms. Due to a large number of simulations becoming extinct before any subsequent farms were detected, we imposed an additional constraint on the selected farms in that they must have purchased at least one animal during the first year of the study period, given that inward movements are a significant risk factor for bTB [16]. In each index case, 5% of the farm's herd was re-assigned from the susceptible to the occult stage of infection given that bTB is endemic but no farms were detected in the first year in which data was available (2008).

5. Evaluation of number of simulations per parameter set

We used Latin Hypercube Sampling (LHS) analysis to evaluate the number of simulations necessary per parameter set to obtain a consistent result [17,18]. We generated 500 sets of the four unknown parameters (ϕ, α, $sens_{sl}$, $sens_{sk}$) through LHS and ran 100 simulations for each parameter set. For each of the three outputs for model assessment, we first identified the parameter set which produced the highest coefficient of variation (CV = sd/mean), when averaged over the 100 simulations, for number of farms infected, number of farms detected, and percent of pairwise distances between infected farms <5 km. Beginning with a single, randomly selected simulation, we incrementally added one simulation at a time, re-calculating the CV after each addition. Convergence can be assumed at the number of simulations in which the CV stabilizes [19]. From this analysis, it was concluded that 75 simulations per parameter set was sufficient for the CV to stabilize (Figure S2). Thus, 75 simulations per parameter set was used for model parameterization.

Figure S2. Changes in the coefficient of variation as additional simulations are performed for a) total number of infected farms, b) number of detected cases, and c) percent of pairwise distances between infected farms that are <5 km.

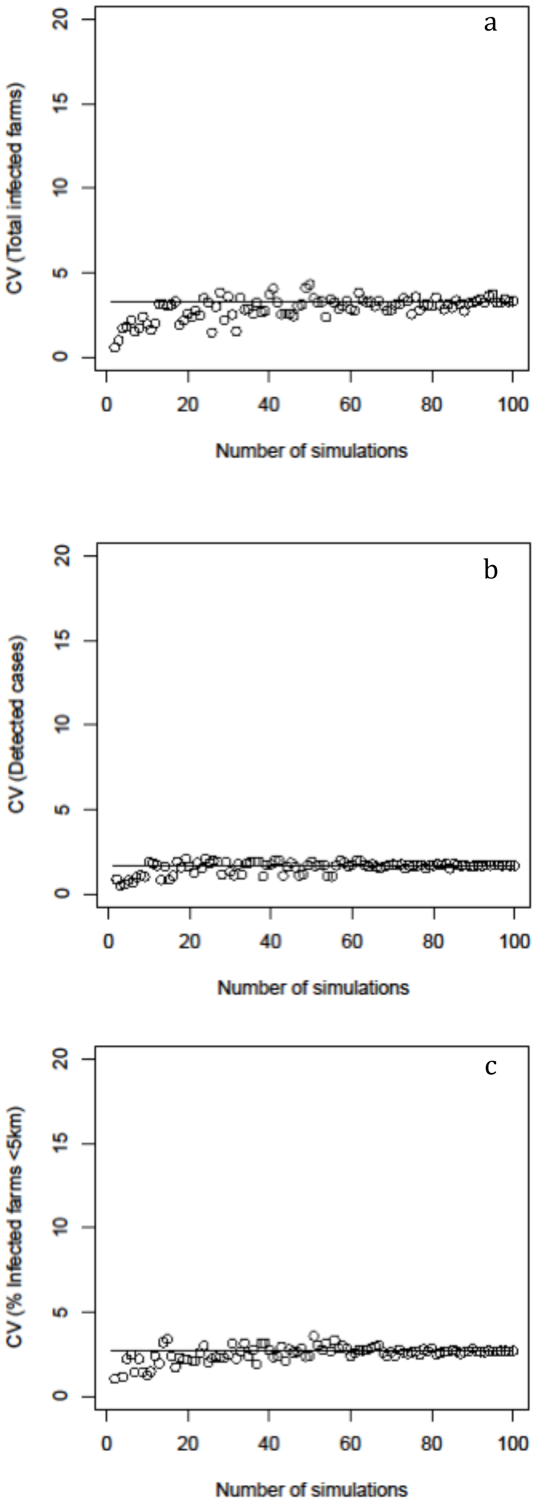

6. Genetic algorithm tuning

A genetic algorithm (GA) was used to optimize parameters in order to minimize the difference between simulated output and observed epidemiological data. In contrast to other model calibration methods, such as Approximate Bayesian Computation (ABC), optimization methods such as GAs are heuristic, machine-learning approaches that produce point estimates of parameters, whereas ABC approximates the posterior distribution of parameters for statistical inference [20,21]. However, the methods share some similarities in that both are likelihood-free and rely on comparisons between simulated and observed data. Furthermore, in the case of the population Monte Carlo and sequential Monte Carlo implementations of ABC, both GA and ABC rely on a population of parameter sets whose propagation is determined via GA-like particle filtering approaches [20,22].

Genetic algorithms require the selection of processes for selection, mutation, and crossover operators. The appropriate processes are situation dependent and must be optimized for the specific model in question. Thus, we performed a series of "tuning experiments" to ensure that the GA could reliably estimate parameters. To achieve this, we used the model to simulate an outbreak with known parameters. We then used the GA to attempt to recover the true parameter values. In the first "experiment," we compared several commonly used crossover operators, including local-arithmetic and blending [23]. The operator that produced parameter estimates most closely aligned to the true parameters was selected and used in the second experiment. The second experiment compared a fitness function based on equal versus variable weighting of each fitness component, and the function which produced estimates most closely aligned to the true parameters was used in subsequent experiments. This process was repeated for the third (selection operators: linear-rank selection, linear-scaling selection, unbiased tournament selection, and selection proportional to Goldberg's sigma truncation scaling), fourth (mutation operators: uniform random mutation, and random mutation around the solution), and fifth (probability of mutation = 0.1 vs 0.2) experiments. Running the GA for 45 generations rather than 30 yielded identical parameter estimates. A GA will produce slightly different results every time it is performed, and it was found that averaging the estimated parameter values from five GA runs produced more accurate parameter estimates than an individual GA run. Therefore, in all experiments and in the final implementation of the GA with real-word data, the GA was performed five times and the parameter estimates were averaged across runs.

The tuned GA utilized a crossover probability of 0.8 with local arithmetic cross over, mutation probability of 0.1 with random mutation around the solution, linear-rank selection, 5% elitism, 30 generations, and a population size of 50 parameter sets per generation. The GA was run for 30 generations, which represented 112,500 individual runs of the model.

To further validate the tuned GA, we ran 1000 simulations of the model with the parameter values estimated by the GA, as well as 1000 simulations using the true parameters. The total number of infected farms, farms detected farms in the final three years (2011-2013), and distribution of pairwise distances between infected farms (proportion of pairwise distances <5, 5-10 km apart) were summarized and compared between simulations using the known and fitted parameter values in order to assess whether the models run with the estimated parameters achieved similar epidemiological dynamics to the true parameters (Figure S3). In general, the fitted and true parameters produced simulations with similar epidemiological dynamics. This analysis, combined with the recovery of all parameter value to within 10% of true values, led us to conclude that the GA was an effective approach for estimated parameters in this system and could be applied to estimate parameters in our real-world data.

Figure S3. Distributions of model outputs using the fitted and true parameter values for a) total number of farms infected, b) number of farms detected from 2011-2013, and proportion of pairwise distances between infected farms that were c) <5 km and d) 5-10 km apart. Red circles represent the "observed" epidemic that was used to fit the model. No "observed" data is presented for panel a given that the true number of infected farms is not observable in real-word data.

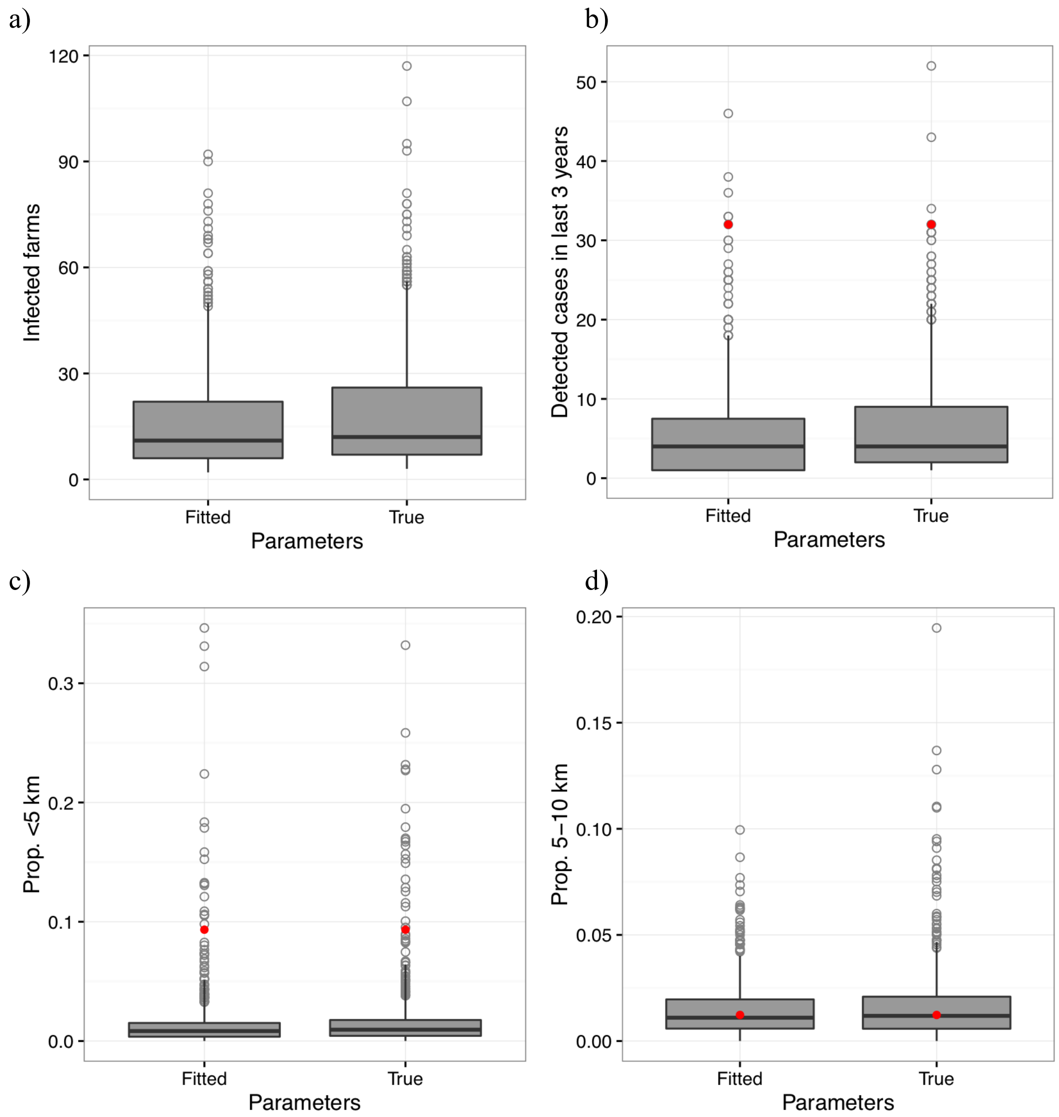

Supplementary Results

Supplementary Table S2: Observed epidemiological data and corresponding predictions from 1000 runs of the fitted model (median, interquartile range (IQR), and 95% prediction interval). Note that in the observed data, detection of farms through annual skin testing versus skin testing as part of contact tracing was not differentiated.

Model output	Observed	Model (median)	Model (IQR)	95% interval
Number of farms infected (including seeds)	unknown	51	40 – 68	30-113
Number of farms detected	58	40	34-48	28 - 72
Number of dairies infected (including seeds)	unknown	30	28-34	25-45
Number of dairies detected	57	28	26-31	24-39
Number of non-dairies infected	unknown	20	11-33	3-74
Number of non-dairies detected	1	11	6-18	2-37
Proportion farms detected at slaughter	0	0.26	0.20 – 0.32	0.10 – 0.42
Proportion farms detected with skin testing	1	0.57	0.48 – 0.66	0.35 – 0.80
Proportion farms detected through tracing		0.16	0.11 – 0.21	0.03 – 0.30
Proportion dairies detected at slaughter	0	0.16	0.12 – 0.21	0.04 -0.33
Proportion dairies detected with skin testing	1	0.81	0.75 – 0.86	0.63 – 0.95
Proportion dairies detected through tracing		0.03	0 – 0.04	0 – 0.12
Proportion non-dairies detected at slaughter	0	.5	0.38-0.57	0 – 0.80
Proportion non-dairies detected with skin testing	1	0	0	0
Proportion non-dairies detected through tracing		0.5	0.40 – 0.62	0.17 – 1.0
Proportion farms detected annually	unknown	0.34	0.19 – 0.46	0.1- 0.68

REFERENCES

1 Álvarez, J. *et al.* Bovine tuberculosis: within-herd transmission models to support and direct the decision-making process. *Res. Vet. Sci.* **97**, S61-68 (2014).

2 Barlow, N. D., Kean, J. M., Hickling, G., Livingstone, P. G. & Robson, A. B. A simulation model for the spread of bovine tuberculosis within New Zealand cattle herds. *Prev Vet Med* **32**, 57-75, doi:Doi 10.1016/S0167-5877(97)00002-0 (1997).

3 Alvarez, J. *et al.* Eradication of bovine tuberculosis at a herd-level in Madrid, Spain: study of within-herd transmission dynamics over a 12 year period. *BMC Vet Res* **8**, 100, doi:10.1186/1746-6148-8-100 (2012).

4 Fischer, E. a. J., van Roermund, H. J. W., Memerik, L., van Asseldonk, M. a. P. & de Jong, M. C. M. Evaluation of surveillance strategies for bovine tuberculosis (*Mycobacterium bovis*) using an individual based epidemiological model. *Prev Vet Med* **67**, 283-301 (2005).

5 Conlan, A. J. K. *et al.* Estimating the hidden burden of bovine turburculosis in Great Britain. *PLOS Compuational Biology* **8**, e1002730 (2012).

6 Rossi, G. *et al.* Epidemiological modelling for the assessment of bovine tuberculosis surveillance in the dairy farm network in Emilia-Romagna (Italy). *Epidemics* **11**, 62-70, doi:10.1016/j.epidem.2015.02.007 (2015).

7 Smith, R. L., Schukken, Y. H., Lu, Z., Mitchell, R. M. & Grohn, Y. T. Development of a model to simulate infection dynamics on *Mycobacterium bovis* in cattle herds in the United States. *J. Am. Vet. Med. Assoc.* **243**, 411-423 (2013).

8 O'Hare, A., Orton, R. J., Bessell, P. R. & Kao, R. R. Estimating epidemiological parameters for bovine tuberculosis in British cattle using a Bayesian partial-likelihood approach. *Proceedings of the Royal Society of London B* **281**, 20140248 (2014).

9 DIEA, E. A. (ed Ministerio de Ganaderia Agricultura Pesca) (Montevideo, Uruguay, 2013).

10 Scotland, A. G. 1-42 (The Scottish Government, Edinburgh, 2011).

11 USDA. 1-39 (Animal and Plant Health Inspection Service, United Stated Department of Agriculture, Fort Collins, CO, 2009).

12 Brooks-Pollock, E., Roberts, G. O. & Keeling, M. J. A dynamic model of bovine tuberculosis spread and control in Great Britain. *Nature* **511**, 228-231 (2014).

13 VanderWaal, K. L. *et al.* Network analysis of cattle movements in Uruguay: Quantifying heterogeneity for risk-based disease surveillance and control. *Prev Vet Med* **123**, 12-22 (2016).

14 Gillispie, D. T. Approximate accerlarated stochastic simulation of chemically reacting systems. *Journal of Chemical Physics* **115**, 1716-1733 (2001).

15 Keeling, M. J. & Rohani, P. *Modelling Infectious Diseases in Human and Animals*. 408 (Princeton University Press, 2008).

16 Picasso, C. *et al.* Epidemiological investigation of bovine tuberculosis outbreaks in Uruguay (2011-2013). *Prev Vet Med* (In press).

17 Martinez, I., Wiegand, T., Camarero, J. J., Batllori, E. & Gutierrez, E. Disentangling the Formation of Contrasting Tree-Line Physiognomies Combining Model Selection and Bayesian Parameterization for Simulation Models. *Am. Nat.* **177**, E136-E152, doi:10.1086/659623 (2011).

18 Thiele, J. C., Kurth, W. & Grimm, V. Facilitating parameter estimation and senstivity analysis of agent-based models: a cookbook using NetLogo and R. *Journal of Artificial Societies and Social Simulation* **17**, 11 (2014).

19 Lorscheid, I., Heine, B. O. & Meyer, M. Opening the 'black box' of simulations: increased transparency and effective communication through the systematic design of experiments. *Comput Math Organ Th* **18**, 22-62, doi:10.1007/s10588-011-9097-3 (2012).

20 Sadegh, M. & Vrugt, J. A. Approximate Bayesian Computation using Markov Chain Monte Carlo simulation: DREAM(ABC). *Water Resources Research* **50**, 6767-6787 (2014).

21 Wu, Q., Smith-Miles, K. & Tian, T. Approximate Bayesian computation schemes for parameter inference of discrete stochastic models using simulated likelihood density. *Bioinformatics* **15 (Suppl 12)**, 1-10 (2014).

22 Akeret, J., Refregier, A., Amara, A., Seehars, S. & Hasner, C. Approximate Bayesian computation for forward modeling in cosmology. *Journal of Cosmology and Astroparticle Physics* **08**, 043 (2015).
23 Scrucca, L. GA: A package for genetic algorithms in R. *Journal of Statistical Software* **53**, 1-37 (2012).
